# Perioperative Care for Children With Syndromic Craniofacial Synostosis Undergoing Le Fort III Surgery: A Retrospective Cohort Study

**DOI:** 10.1097/SCS.0000000000010400

**Published:** 2024-06-10

**Authors:** Andrea Restivo, Egle Rondelli, Marco Giani, Mattia Moretti, Chiara Fossati, Roberto Rona, Alessandra Moretto, Andrea Biondi, Fabio Mazzoleni, Giuseppe Foti

**Affiliations:** * Department of Medicine and Surgery, Università degli Studi di Milano-Bicocca; † Department of Emergency and Intensive Care, Fondazione IRCCS San Gerardo dei Tintori; ‡ Department of Neuroscience, Maxillofacial Surgery, Fondazione IRCCS San Gerardo dei Tintori; § Department of Pediatrics, Fondazione IRCCS San Gerardo dei Tintori, Monza, Italy

**Keywords:** Complications, craniofacial synostosis, Le Fort III osteotomy, multidisciplinary approach, perioperative management

## Abstract

**Objective:**

To present characteristics, surgical variables, complications, and postoperative care in pediatric patients with craniofacial synostosis undergoing Le Fort III osteotomy.

**Background:**

Craniofacial synostoses are a group of genetic syndromes that result in premature fusion of cranial and facial sutures, leading to craniofacial deformities and associated complications. Midface advancement through Le Fort III osteotomy is the most frequent surgical option for these conditions.

**Methods:**

Retrospective monocentric cohort study including patients with syndromic craniofacial synostosis who underwent Le Fort III osteotomy between 2009 and 2022 in a specialized referral center. Data collection encompassed surgical time, blood loss, intraoperative transfusions, fluid balance, and postoperative parameters such as duration of invasive mechanical ventilation and intensive care unit (ICU) length of stay.

**Results:**

Twenty-six children were included in the analysis. The median surgical time was 345 minutes (300–360), with an estimated blood loss of 15 (9.9–24) mL/kg. Patients required a median transfusion of 12.63 (7.1–24.5) mL/kg of packed red blood cells and 19.82 (11.1–33) mL/kg of fresh frozen plasma. Intraoperative fluid balance was + 12.5 (0.8–22.8) mL/kg, with a median infusion of 30.4 (23.9–38.7) mL/kg of crystalloids. All patients were transferred to the ICU after surgery to ensure a safe environment for extubation. The median duration of mechanical ventilation in the ICU was 30 (20.25–45) hours, and postoperative ICU length of stay was 2 (2–4) days, and complications were infrequent, with only one extubation failure recorded.

**Conclusion:**

Le Fort III osteotomy in craniofacial synostosis patients may be characterized by a complex perioperative course. A multidisciplinary approach in the care of these patients allows for minimizing complications in the perioperative phase. Further research is needed to enhance perioperative management in this unique patient population.

Craniofacial synostosis is a group of syndromes caused by genetic mutations in fibroblast growth factor receptors or the *TWIST* gene. These conditions are characterized by the premature fusion of cranial and facial sutures, leading to deformities in the skull and skull base, as well as midface hypoplasia and retrusion.^1^ These morphological changes can result in complications such as increased intracranial pressure, exorbitism, upper airway obstruction, obstructive sleep apnea syndrome (OSAS), and craniofacial dysmorphism.^2^ The continuum of care for individuals with craniofacial synostosis is typically complex and involves various surgical procedures throughout their growth, including cranioplasty, Le Fort III osteotomy, orthognathic surgery, and ancillary procedures.^3^ The midfacial advancement plays a key role in the therapeutic approach to craniofacial synostosis, as it corrects the main feature of these syndromes, improving ocular, respiratory, and esthetic problems, and it can be done with fronto-orbital monobloc procedure or Le Fort III osteotomy. However, the midface advancement through Le Fort III osteotomy is the most frequent option. It has a great technical complexity, which is reflected in the challenge represented by perioperative care.^4,5^ Airway management and blood loss represent the key issues during surgery, whereas pain control and possible extubation difficulties may require intensive care unit (ICU) admission to avoid or promptly manage any complications.^6,7^

The aim of the study is to retrospectively describe the perioperative management, the rate of complications, and short and long-term outcomes in patients affected by craniofacial synostosis who underwent Le Fort III osteotomy at our institution, a referral center for craniosynostosis.

## METHODS

We performed a retrospective study including patients affected by craniofacial synostosis who underwent Le Fort III osteotomy between 2009 and 2022 at Fondazione IRCCS San Gerardo dei Tintori.

### Patient Management

Our institution gathers cases of craniosynostosis from the whole Italian territory. Preoperative surgical planning is a collaborative effort involving a multidisciplinary team that includes maxillofacial surgeons, a dedicated pediatric anesthesia team (comprising a physician and nurse), intensivists, neurosurgeons, child neuropsychiatrists, ear, nose, and throat physicians, pediatric cardiologists, and pediatric ophthalmologists.

For airway management, direct laryngoscopy is typically the primary choice for intubation. Alternative devices, such as fiberscopes and video laryngoscopes, are also available for managing both predicted and unpredicted difficult airways. Armored endotracheal tubes are utilized. Anesthesia is maintained with halogenated gas and a continuous infusion of remifentanil. Intraoperative monitoring encompasses various parameters, including peripheral oxygen saturation, electrocardiography, end-tidal CO_2_, invasive blood pressure through an arterial line, temperature, and diuresis through a urinary catheter. In addition, tranexamic acid is administered as a bolus (typically between 10 mg/kg and 20 mg/kg) and through continuous infusion (ranging from 10 to 20 mg/kg/h) throughout the surgical procedure to reduce blood loss and the need for transfusions.^8,9^ Arterial blood is sampled to monitor gas exchange, hemoglobin levels, platelet count, and standard coagulation tests, including prothrombin time, activated partial thromboplastin time, and fibrinogen levels. In addition, in cases of severe bleeding, thromboelastography is performed to promptly detect and address specific coagulation abnormalities.

After the surgical procedure, children are admitted to our mixed adult-pediatric ICU to prevent and, if necessary, treat postoperative complications. Extubation typically takes place the day after the surgery.

### Data Collection

We collected information regarding perioperative management and complications. Specifically, we recorded data on surgical time, preoperative hemoglobin levels, the Mallampati score during preoperative evaluation, the Cormack-Lehane grading at intubation, the intraoperative use of tranexamic acid, cumulative fluid balance, and estimated blood loss at the end of the surgical procedure, as well as total blood and/or plasma transfusion. In addition, we gathered data related to postoperative ICU admission, including the length of stay, duration of invasive ventilation, the modality of oxygen therapy or respiratory support after extubation, fluid balance on the first day of admission, cumulative fluid balance at ICU discharge, and the use of inhaled adrenaline and/or helium after extubation, as well as the use of steroids before and after extubation, and blood transfusions.

Continuous data are presented as median and 25th to 75th percentile, and categorical data as count and percentage.

## RESULTS

In the study period, a total of 26 children underwent craniofacial surgery for syndromic craniosynostosis and were included in this study. Supplemental Table S1 (Supplemental Digital Content Table S1, http://links.lww.com/SCS/G407) shows the baseline characteristics of the study population.

In our study population, the surgical indication for Le Fort III surgery included 1 patient with a preexisting tracheostomy, 12 patients with moderate to severe OSAS, and 13 patients solely for correcting exorbitism and skeletal deformities. The median Mallampati score during the anesthesiologist’s preoperative evaluation was 2 (1.25–2). At the laryngoscopy, the median degree of the Cormack-Lehane classification was 1 (1–2). Intubation was performed in 92% of cases by direct laryngoscopy. In 2 cases (4%), alternative methods were necessary: one case involved the use of a video laryngoscope (4%) and the other case required fiberoptic intubation (4%). All 25 (100%) patients were intubated with an armored endotracheal tube. The median surgical time was 345 minutes (300–360 min). The estimated blood losses during the intraoperative period were 435 (300–612.5) mL, and required transfusion of 350 (254–480) mL (12.6 mL/kg; 7.2–23.2) of packed red blood cells and 500 (400–587.5) mL of fresh frozen plasma (19.82 mL/kg; 11.46–31.66) during the surgical procedure. A bolus of tranexamic acid 500 mg (500–1000 mg), followed by a continuous infusion of 5 mg/kg/h for 6 hours, was administered to a total of 15 patients (60%). The intraoperative fluid balance was +270 (50–680) mL, with a median infusion of 1000 mL of crystalloids (450–1300). A total of 10 (40%) patients were infused with 390 mL of colloids (200–912.5 mL) in addition to crystalloid fluid therapy. As for the surgical technique, in our sample, the average advancement of the A point was 15.5 mm, ranging from 4 to 24 mm. This advancement was achieved in 18 cases through distraction osteogenesis and the utilization of a rigid external device, whereas the remaining 8 patients underwent rigid fixation using titanium plates and screws. Supplemental Table S2 (Supplemental Digital Content Table S2, http://links.lww.com/SCS/G407) presents intraoperative data and outcomes, stratified according to the clinical syndrome. Children with Apert syndrome received a significantly higher volume of crystalloid compared with those with Crouzon or Pfeiffer syndrome.

**FIGURE 1:**
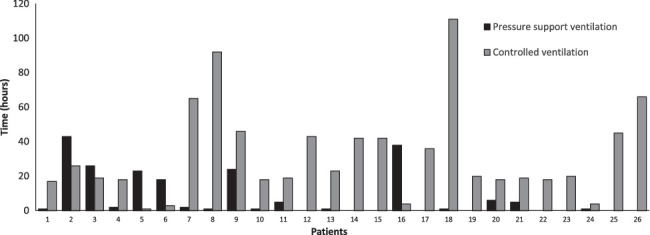
Mechanical ventilation modes during the weaning process. The figure displays the time spent on controlled ventilation versus pressure support ventilation for each patient during the weaning process from mechanical ventilation.

As for the postoperative phase, the median ICU length of stay in ICU was 2 days (2–4), and mechanical ventilation lasted 30 hours (20–45). 9 patients (34.6%) were extubated directly at the recovery of spontaneous respiratory drive, whereas the remaining patients (65.4%) spent a median of 3.5 hours (1–22 h) in pressure support ventilation during the weaning from invasive mechanical ventilation. Figure 1 provides further details on the duration spent on controlled mechanical ventilation compared with pressure support ventilation.

Median FiO_2_ before extubation was 30% (30–35), with a ratio of arterial oxygen tension to inspired oxygen fraction (pO_2_/FiO_2_) of 420 (343–449). After extubation, oxygen was delivered to the patients using Venturi Masks in 13 cases (50%), high-flow nasal cannula in 2 cases (7.7%), and low-flow oxygen therapy devices in 3 cases (11.5%). Eight patients (30.8%) did not require oxygen therapy. The median administered FiO_2_ after extubation was 40% (21–40). Corticosteroid therapy was used in 22 patients (84.6%) before extubation and in 21 patients (80.8%) after extubation. A total of 5 patients (19.2%) received inhaled helium therapy, with a median inspiratory concentration of 60% (60–60). Inhaled adrenaline was administered to 7 (26.9%) patients after extubation.

Extubation failure occurred in only one case out of 26 (3.84%). The affected child, who was the youngest patient at 2 years old and underwent surgery due to severe OSAS and eyelid ptosis, required reintubation. This patient had a prolonged hospitalization in the ICU lasting 58 days due to moderate acute respiratory distress syndrome, infective complications, and neurological issues. These complications hindered respiratory weaning and necessitated a surgical tracheostomy. Eventually, after an extended stay in the ICU, the child was successfully weaned from mechanical ventilation and was discharged with a complete recovery of neurological and respiratory functions.

The median value of fluid balance in the first ICU day was + 140 mL (−75; +275 mL). The cumulative fluid balance during the entire ICU stay had a median value of +60 mL, but exhibited a broad interquartile range (−1100; +600 mL). Diuretic therapy was required in 11 patients (42.3%) to manage their fluid balance. The median blood losses from the surgical cranial drainage were 117.5 mL (85–187.5 mL). Only 5 patients (19.23%) required blood transfusions during their ICU stay. Two patients (7.7%), including the previous case, experienced infectious complications and required antibiotic therapy.

As for the surgical outcome, patients with OSAS demonstrated a substantial improvement in their condition, with a decrease in the Apnea-Hypopnea Index from 20.2/h (5.9–42.3/h) to 3.0/h (0.8–4.1/h). Furthermore, in the patient with a preexisting tracheostomy, successful removal of the tracheostomy tube was achieved. Furthermore, patients exhibited a noticeable esthetic enhancement, attributed to the significant advancement of the midface.

## DISCUSSION

In this retrospective cohort study, we have comprehensively described the perioperative management, complication rates, and outcomes of children with syndromic craniosynostosis who underwent Le Fort III osteotomy at our tertiary referral hospital. Our findings shed light on crucial aspects of perioperative care, including surgical approaches, intraoperative management, and postoperative results. It is noteworthy that all patients in our study had syndromic craniosynostosis, with Crouzon and Apert syndromes being the most prevalent. The successful perioperative management of children with syndromic craniosynostosis underscores the pivotal role of a multidisciplinary team in preventing complications and effectively addressing the unique challenges associated with these patients.

The literature on perioperative management of children with craniosynostosis is notably limited Identified risk factors that extend ICU stays after craniosynostosis surgery encompass issues such as blood loss, the age of the patient at the time of surgery (especially those below 5 y old), and the occurrence of infections.^10^ While there exists a significant body of literature comparing various surgical approaches for rectifying midface hypoplasia in syndromic craniosynostosis, along with their associated surgical complications,^11,12^ it is worth noting that there is a paucity of studies specifically focusing on the perioperative management of these patients.

The Pediatric Craniofacial Collaborative Group (PCCG) conducted the largest and most significant study in this field,^13^ which encompassed 49 patients undergoing Le Fort III surgery. Crouzon syndrome was the most prevalent syndrome, accounting for 45% of cases. Comparatively, the median age and weight of the patients in the PCCG study were slightly higher than those in our population. The median duration of surgery (366 min) closely aligned with the findings in our study (345 min). However, it is noteworthy that the median length of ICU stays in the PCCG study (4 d; 3–5.75) exceeded that observed in our cohort (2 d). Concerning the extubation time, the approach of the PCCG group closely mirrored ours, with 73% of patients being extubated 24 hours after surgery (compared with 69.2% in our study). In terms of intraoperative blood and plasma transfusions, the study conducted by Glover and colleagues reported a higher median volume of red blood cell transfusions (17 ± 15 mL/kg) compared with our study (12.6 ± 16 mL/kg). Conversely, the volume of fresh frozen plasma transfusions was lower in their study (15.1 ± 7.6 mL/kg) compared with our study (19.8 ± 20.2 mL/kg). Of patients, 16% in their study required blood transfusions in the postoperative period, compared with 19.2% of our population. While analyzing our data on transfusions, it appears that intraoperative blood losses might have been underestimated, as the transfusion volume (350 mL for packed red blood cells and 500 mL fresh frozen plasma) exceeded the estimated losses (450 mL). In addition, the median hemoglobin value upon admission to the ICU was 1.7 g/dL lower compared with preoperative levels, which supports the hypothesis of an underestimation of intraoperative blood loss.

It is important to note that our study has certain limitations. Firstly, it follows a retrospective design. Secondly, the sample size was relatively small. However, it is essential to consider that syndromic craniosynostosis is a rare disease, and to the best of our knowledge, our report represents the largest single-center study ever conducted on the perioperative anesthesiologist and intensive care management of these patients.

## CONCLUSION

The adoption of a multidisciplinary and comprehensive approach in the care of children with syndromic craniosynostosis is crucial for ensuring safe perioperative management while minimizing complications. The complexity of these cases calls for ongoing research and larger studies to identify potential areas for improvement in the perioperative care of these patients.

## Supplementary Material
