# Supplementary material for: Perioperative Care for Children With Syndromic Craniofacial Synostosis Undergoing Le Fort III Surgery: A Retrospective Cohort Study
Source: J Craniofac Surg. 2024 Jun 10;35(6):1692–5. doi: 10.1097/SCS.0000000000010400 (PMC11346705; doi:10.1097/SCS.0000000000010400)
Supplement: Supplementary file 1 [file scs-35-1692-s001.docx]

**Supplementary Digital Content**

**Table S1:** Baseline characteristics of the study population.

|  |  |
| --- | --- |
| Age, Years | 9 (5.5-12.5) |
| Weight, kg | 27 (18.5-42) |
| Preoperative Hb level, g/dl | 12.9 (12-13.5) |
| Syndrome  - Apert  - Crouzon  - Pfeiffer | 9 (33.3%)  11 (40.7%)  6 (22.2%) |

Data are presented as median (25th-75th percentile) or as count and percentage. Hb, hemoglobin.

**Table S2.** Characteristics of the study population stratified according to the clinical syndrome.

|  | Apert  syndrome (n.9) | Crouzon syndrome(n.11) | Pfeiffer  syndrome(n.6) |
| --- | --- | --- | --- |
| *Baseline* |  |  |  |
| Age, years | 9 (515-1315) | 11 (6-14) | 415 (2-7) |
| Weight, kg | 35 (19.5-45) | 27 (20-45) | 13.5 (9.75-29) |
| Mallampati score | 2 (2-2) | 1.5 (1-2) | 2 (2-2) |
| *Operating Room* |  |  |  |
| Cormack-Lehane grade | 1 (1-1.5) | 1 (1-2) | 2 (1.5-2) |
| Hb level before surgery, g/dL | 13.3 (12.45-14.1) | 12.9 (11.5-13.5) | 12.1 (11.125-13) |
| Hb level at ICU admission, g/dL | 10.6 (8.7-12) | 11 (10.2-12.1) | 10.4 (9.75-12.125) |
| Hb variation after surgery, g/dL | -3.3 (-4.5 - -1.4) | -1.4 (-2.9 - -1) | -1.5 (-2.9 - +0.3) |
| Intraoperative fluid balance, mL/kg | 16.9 (5.9-38.9) | 6 (-3-19.4) | 17.3 (-1.8-23) |
| Estimated blood loss, mL/kg | 18.6 (10.5-34.2) | 15 (7.4-20) | 15 (14.6-26.8) |
| Intraoperative crystalloid infusion, mL/kg | 50 (35-71) | 26 (22-32) **$** | 22 (10-32) **$** |
| Intraoperative colloid infusion, mL/kg | 12 (0-20) | 0 (0-5) | 0 (0-16) |
| Intraoperative PRBC transfusion, mL/kg | 9.6 (7.1-20.9) | 10.4 (5.6-20) | 25.1 (14.4-47.7) |
| Intraoperative FFP transfusion, mL/kg | 14.3 (0-28.1) | 19.6 (11.1-25) | 34.6 (23.6-51.3) |
| *ICU* |  |  |  |
| PaO_2_/FiO2 before extubation, mmHg | 410 (307.5-457) | 428 (360-450) | 415 (311-502.5) |
| ICU cumulative fluid balance, mL/kg | 6 (-32 - 44) | -13.8 (-42 - 15.2) | 11.7 (-54 - 41) |
| Duration of IMV in the ICU, hours | 42 (18-68) | 24 (20-42) | 33.5 (23-57) |
| ICU stay, days | 3 (2-4) | 2 (2-4) | 2 (2-17.5) |

$=p<0.05 vs Apert syndrome. ICU, intensive care unit; PRBC, packed red blood cells; FFP, fresh frozen plasma; PaO2/FiO2, ratio of arterial oxygen tension to inspiratory oxygen fraction; IMV, invasive mechanical ventilation.
